# ChatGPT Conquers the Saudi Medical Licensing Exam: Exploring the Accuracy of Artificial Intelligence in Medical Knowledge Assessment and Implications for Modern Medical Education

**DOI:** 10.7759/cureus.45043

**Published:** 2023-09-11

**Authors:** Fahad K Aljindan, Abdullah A Al Qurashi, Ibrahim Abdullah S Albalawi, Abeer Mohammed M Alanazi, Hussam Abdulkhaliq M Aljuhani, Faisal Falah Almutairi, Omar A Aldamigh, Ibrahim R Halawani, Subhi M K. Zino Alarki

**Affiliations:** 1 Department of Plastic Surgery, King Abdullah Medical City, Makkah, SAU; 2 College of Medicine, King Saud Bin Abdulaziz University for Health Sciences, Jeddah, SAU; 3 College of Medicine, Tabuk University for Health Sciences, Tabuk, SAU; 4 Department of Pediatrics, Faculty of Medicine, University of Tabuk, Tabuk, SAU; 5 Faculty of Medicine, Ibn Sina National College, Jeddah, SAU; 6 College of Medicine, Unaizah College of Medicine and Medical Sciences, Qassim University, Unaizah, SAU; 7 College of Medicine, King Faisal University, Dammam, SAU; 8 Faculty of Medicine, King Abdulaziz University, Jeddah, SAU; 9 Department of Surgery, King Khalid University Hospital, Riyadh, SAU

**Keywords:** standardized medical exam, ai in healthcare, smle, saudi medical licensing exam, medical education, artificial intelligence, chatgpt-4

## Abstract

Background

The application of artificial intelligence (AI) in education is undergoing rapid advancements, with models such as ChatGPT-4 showing potential in medical education. This study aims to evaluate the proficiency of ChatGPT-4 in answering Saudi Medical Licensing Exam (SMLE) questions.

Methodology

A dataset of 220 questions across four medical disciplines was used. The model was trained using a specific code to answer the questions accurately, and its performance was assessed using key performance indicators, difficulty level, and exam sections.

Results

ChatGPT-4 demonstrated an overall accuracy of 88.6%. It showed high proficiency with *Easy* and *Average* questions, but accuracy decreased for *Hard* questions. Performance was consistent across all disciplines, indicating a broad knowledge base. However, an error analysis revealed areas for further refinement, particularly with category (Option) A questions across all sections.

Conclusions

This study underscores the potential of ChatGPT-4 as an AI-assisted tool in medical education, demonstrating high proficiency in answering SMLE questions. Future research is recommended to expand the scope of training and evaluation as well as to enhance the model’s performance on complex clinical questions.

## Introduction

OpenAI introduced ChatGPT, a comprehensive large language model (LLM), to the public in November 2022. Often referred to as GPT-3.5, this model underwent training on vast text datasets using both supervised and unsupervised learning methods and then refined using reinforcement learning based on human input. A distinguishing feature of ChatGPT is its inability to browse the web, setting it apart from other chatbots with internet access. While OpenAI’s proprietary version of ChatGPT has online querying capabilities, the public version does not currently offer this feature, though it is anticipated in forthcoming releases. On March 14, 2023, OpenAI unveiled GPT-4, another LLM trained in a manner consistent with its previous model [1]. ChatGPT, along with a host of other chatbots, has captured both public attention and academic curiosity. This new wave of artificial intelligence (AI)-driven technology holds promise in bringing transformative changes to healthcare and the realm of medical education [2-4]. Following its launch, ChatGPT has become a topic of conversation and study among medical education communities. Gilson et al. highlighted that ChatGPT could attain scores comparable to a third-year US medical student. They further underscored ChatGPT’s capacity for logical and informative feedback in the majority of its replies. This indicates that ChatGPT holds potential as an engaging medical education instrument to bolster learning [5].

Lately, several teams have assessed ChatGPT’s efficacy on national medical licensing tests, encompassing exams from countries such as China, Japan, and Germany [6-8]. Although these discoveries could signal a paradigmatic change in the creation and assessment of medical exam questions, the depth of their scientific impact is still unclear. Most of what we know about ChatGPT’s ability to tackle tests comes from single-country studies that evaluate a diverse and sometimes non-representative selection of questions. Up to now, there has been a noticeable lack of research offering a cross-national perspective and contrasting ChatGPT’s performance in medical exams using a stringent approach.

The Saudi Medical Licensing Exam (SMLE) is a comprehensive evaluation mechanism implemented by the Saudi Commission for Health Specialties to qualify physicians, whether domestic or international, to practice medicine in Saudi Arabia, or to permit postgraduate medical students to apply for local residency programs. It is a compulsory examination for all prospective doctors during their internship as part of their bachelor’s degree. The exam’s structure was created by the SMLE steering committee, which includes members from the Saudi Medical College Deans. The exam is designed to test a specific learning domain, encompassing not only knowledge but also cognitive abilities (such as interpretation, analysis, decision-making, reasoning, and problem-solving) and attitude [9]. The SMLE is a standardized assessment program designed to evaluate the knowledge and skills of medical graduates seeking to practice in Saudi Arabia [10]. By generating qualitative and quantitative feedback, our research strives to shed light on the potential and viability of ChatGPT’s integration into the healthcare system.

The objective of this study is to evaluate the performance of ChatGPT-4, a state-of-the-art AI language model developed by OpenAI, in answering questions from the SMLE. This assessment aims to understand the model’s accuracy, proficiency, and reliability in interpreting and responding to complex medical questions that are used to gauge the competency of medical graduates in Saudi Arabia. The results from this study will offer insights into the potential applications of using advanced AI models such as ChatGPT-4 in medical education and testing.

## Materials and methods

This study was designed to evaluate the efficacy of the ChatGPT-4 model in answering multiple-choice questions (MCQs) with four options, i.e., A, B, C, and D, from the SMLE.

Data collection

A total of 220 questions were extracted from the SMLE test bank available on the CanadaQBank website (https://canadaqbank.com/smle.php). This dataset was accessed on July 9, 2023. Four researchers collaborated in the data collection process to ensure comprehensive coverage of questions and eliminate potential bias in question selection. The questions were derived from various sections of the SMLE, including Medicine, Pediatrics, Obstetrics and Gynecology, and Surgery, to ensure diversity in subject matter. Each question was accompanied by four potential answers (labeled A, B, C, D), with only one correct response.

We selected a total of 220 questions for evaluation, mirroring the structure of the actual (SMLE). This decision was made to ensure that our study accurately reflected the conditions and challenges of the real examination. Furthermore, the sections from which we distributed were as follows: 30% of the questions were from Medicine, 25% were from Obstetrics and Gynecology, 25% were from Pediatrics, and the remaining 20% were from Surgery. The questions were chosen based on the guidelines provided in the Commission’s handbook [10]. This approach allowed us to maintain the diversity and complexity of the SMLE, thereby providing a comprehensive assessment of the capabilities of the ChatGPT-4 model in a realistic exam scenario.

Model training and evaluation

The ChatGPT 4.0 model was trained using the given codes, which defined its role as a medical professional with the knowledge required to answer SMLE questions accurately and coherently. We gave ChatGPT 4.0 the following training code: “You are a medical professional, I will give you Saudi Medical License Exam (SMLE) questions and four multiple choice for each question. You have the knowledge required to choose one single answer in an accurate, coherent manner. I will provide you with the questions from multiple tests on various topics such as medicine, surgery, obstetrics and gynecology, and pediatrics. I will provide you with the questions and I want you to choose the correct answer.” Without this training code, the ChatGPT 4.0 model would not be able to answer the questions accurately. Instead, it might default to its general behavior, which includes referring the user to a specialist doctor for consultation. This training code is crucial as it provides the model with specific information about the nature of the questions and their source, and instructs it to select one correct answer from the four options provided. This prompt provided the model with information about the nature of the questions and their source. It also directed the model to select one correct answer from the four options provided.

Following the training phase, the model was evaluated on its ability to select the correct answer for each question. Performance metrics, such as accuracy, precision, and recall, were computed to assess the model’s overall performance. The model’s performance was further evaluated across different question difficulty levels and sections.

Statistical analysis

Data analysis was performed using SPSS, version 26.0 (IBM Corp., Armonk, NY, USA). Given the descriptive nature of our study, we primarily focused on descriptive statistics to gain insights from our dataset. Specifically, frequencies and percentages were calculated for all categorical variables to offer a comprehensive understanding of the distribution and characteristics of the data.

We calculated key performance indicators to gauge the model’s performance. The metrics we focused on included accuracy and precision for each response category (A, B, C, D). Accuracy was determined as the proportion of total correct answers provided by the model out of all the questions it attempted. This gave us a general sense of how often the model was correct in its predictions. Precision, on the other hand, was calculated for each category. It represented the ratio of true positives (i.e., correct answers for a particular category) to all instances that the model classified as positive for that category. This gave us an understanding of how precise the model was in its predictions for each specific category.

Furthermore, an error analysis was undertaken to discern the types and frequencies of mistakes made by the model. This was crucial in pinpointing the specific areas that might benefit from further refinement. We also evaluated the model’s performance based on the difficulty level of the questions. We computed the mean accuracy for the *Easy*, *Average*, and *Hard* question categories. This analysis was intended to discern any possible correlation between the intricacy of a question and the model’s propensity to answer it correctly. Finally, the performance was assessed according to the different sections of the SMLE. This analysis revealed how well the model responded to questions related to Medicine, Pediatrics, Obstetrics and Gynecology, and Surgery, thus providing an indication of its strengths and areas for improvement in different medical disciplines. Through these detailed statistical analyses, a comprehensive understanding of ChatGPT-4’s capabilities in answering SMLE questions was attained.

## Results

We utilized a dataset comprising 220 questions, divided as per the SMLE blueprint, to evaluate the performance of the ChatGPT-4 model. The results of this evaluation are organized by key performance indicators, performance across different levels of question difficulty, and performance based on the section of the exam.

ChatGPT’s key performance indicators

**Figure 1:**
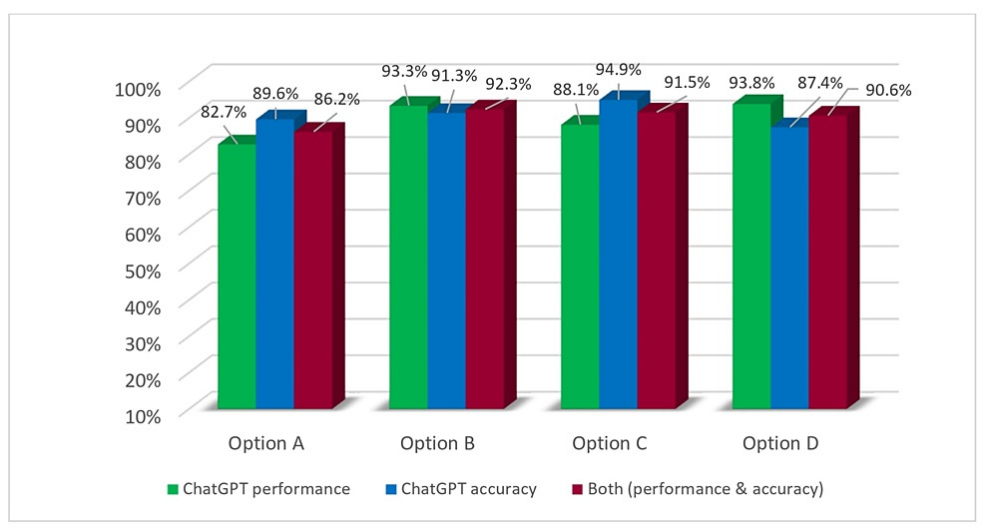
ChatGPT’s performance via answer options.

The overall success rate of ChatGPT-4 in correctly answering the questions was found to be 88.6%. We also measured how well ChatGPT-4 did for each question category (options), A, B, C, and D. The performance of the ChatGPT-4 model was evaluated across four response options (A, B, C, D). For option A, out of 52 instances, the model correctly answered 43 (82.7%) questions and incorrectly answered nine (17.3%). In the case of option B, out of 45 instances, the model correctly answered 42 (93.3%) questions and incorrectly answered three (6.7%). For option C, out of 42 instances, the model correctly answered 37 (88.1%) questions and incorrectly answered five (11.9%). Lastly, for option D, out of 81 instances, the model correctly answered 76 (93.8%) questions and incorrectly answered five (6.2%). These results provide a comprehensive understanding of the model’s performance across the different response categories (Figure 1).

Performance based on difficulty level

**Figure 2:**
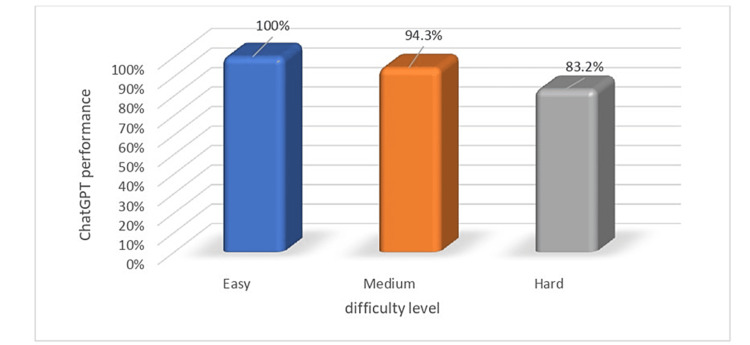
ChatGPT performance via questions difficulty levels.

We next examined how ChatGPT performed for questions of different difficulty levels. To ascertain the difficulty level of each question, we turned to the historical performance metrics provided by the CanadaQBank website. For every question within their database, CanadaQBank not only indicates the proportion of users who answered the question correctly but also categorizes the questions based on this success rate. This categorization, rooted in real-world performance, serves as a reflection of each question’s complexity. CanadaQBank’s method ensures that the difficulty categorization of each question is based on actual user performance, eliminating subjective biases and offering a consistent, objective measure of a question’s challenge level. For *Average* (medium) difficulty-level questions, out of 53 instances, the model correctly answered 50 questions, resulting in a success rate of 94.3%. It incorrectly answered three questions, yielding an error rate of 5.7%. For *Easy* difficulty-level questions, the model showed exceptional performance. Out of 54 instances, it correctly answered all questions, achieving a success rate of 100%. For *Hard* difficulty-level questions, out of 113 instances, the model correctly answered 94 questions, achieving a success rate of 83.2%. It incorrectly answered 19 questions, resulting in an error rate of 16.8% (Figure 2).

Performance based on exam section

**Figure 3:**
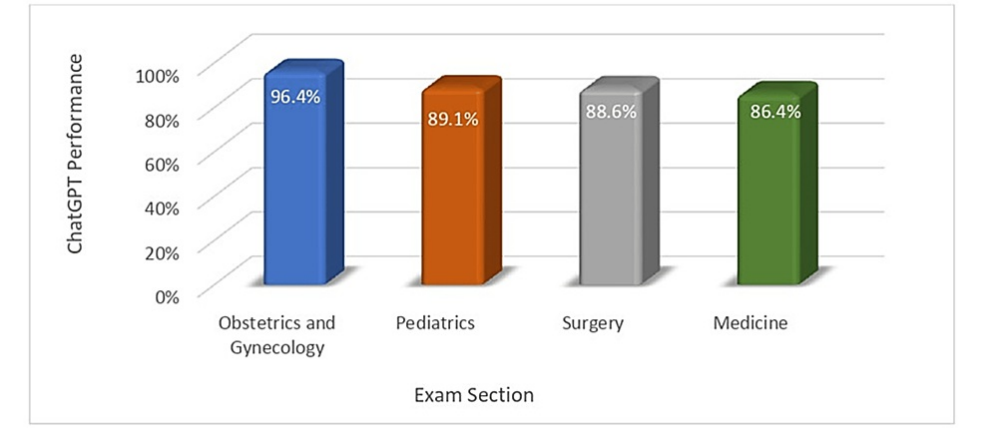
ChatGPT performance across the exam sections.

We then examined how well ChatGPT-4 did based on the exam section the questions came from. The model did well across all sections. In the Medicine section, out of 66 instances, the model correctly answered 57 questions. It incorrectly answered nine questions, yielding an error rate of 13.6%. In the Obstetrics and Gynecology section, out of 55 instances, the model correctly answered 53 questions. It incorrectly answered two questions, resulting in an error rate of 3.6%. In the Pediatrics section, out of 55 instances, the model correctly answered 49 questions. It incorrectly answered six questions, yielding an error rate of 10.9%. In the Surgery section, out of 44 instances, the model correctly answered 39 questions. It incorrectly answered five questions, yielding an error rate of 11.4% (Figure 3).

Error analysis

Finally, we examined the types of errors made by ChatGPT-4. An error analysis was conducted to identify the types and frequencies of errors committed by the ChatGPT-4 model across different sections and response categories.

**Figure 4:**
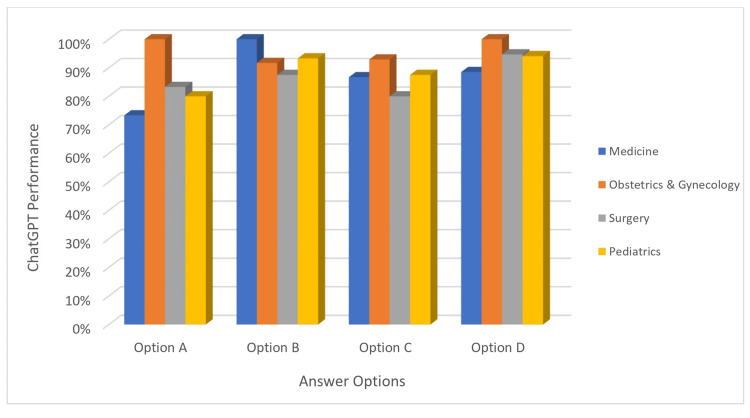
ChatGPT performance across the exam sections and answer options.

In the Medicine section, the model made errors primarily in category A, with an error rate of 26.7%. Categories C and D also saw error rates of 13.3% and 11.5%, respectively. Category B had no errors. In the Obstetrics and Gynecology section, the model made a few errors in categories B and C, with error rates of 8.3% and 7.1%, respectively. There were no errors in categories A and D. In the Pediatrics section, the model made the most errors in category A, with an error rate of 20%. Categories B, C, and D saw fewer errors, with rates of 6.7%, 12.5%, and 5.9%, respectively. In the Surgery section, the model made errors primarily in category A, with an error rate of 16.7%. Categories B and C also saw errors, with rates of 12.5% and 20.0%, respectively. Category D had a low error rate of 5.3% (Figure 4).

This error analysis provides insights into specific areas where the model could benefit from further refinement. It appears that the model struggles the most with category A questions across all sections, suggesting that future training could focus on improving performance in this area.

## Discussion

Our findings demonstrate the substantial potential of ChatGPT in assisting medical education, particularly in the context of preparing for exams such as the SMLE. This study presents a comprehensive evaluation of ChatGPT’s performance on a set of 220 SMLE questions, covering four major medical disciplines, namely, Medicine, Pediatrics, Obstetrics and Gynecology, and Surgery.

The overall accuracy of the model was 88.6%, showing a high degree of proficiency in answering the SMLE questions correctly. While our study showcases the high potential of ChatGPT-4 as a supplementary educational tool, the margin of error of 4-16% emphasizes that it should be used cautiously and in conjunction with traditional study methods and expert consultation. Given the present limitations, ChatGPT-4 may not yet be ready for standalone use in high-stakes exam preparation or medical decision-making. However, its high level of accuracy in answering *Easy* and *Average* questions suggests that it could be a valuable asset for foundational learning and ongoing review. As we continue to refine and train the model, we anticipate that its applicability in medical education and assessment will broaden, eventually revolutionizing the way we prepare for exams and engage in lifelong learning.

Our observations are consistent with the findings of Kung et al. [11], who conducted a similar evaluation of ChatGPT’s performance but focused on the United States Medical Licensing Examination (USMLE). In their study, they demonstrated that ChatGPT was capable of passing the USMLE, thereby affirming the substantial potential of AI in medical education and assessment. What sets our study apart is the focus on the SMLE, offering a unique perspective on how ChatGPT performs across different standardization bodies and in different cultural and educational contexts. Additionally, while Kung et al. concentrated on the pass/fail metrics, our study provides a detailed error analysis to identify specific areas where ChatGPT could benefit from further refinement. An intriguing pattern emerged from the analysis of ChatGPT’s performance based on the difficulty level of questions. The model was remarkably accurate with *Easy* and *Average* questions, suggesting a strong grasp of foundational medical knowledge. However, the accuracy decreased for *Hard* questions, indicating that the model might benefit from additional training on complex clinical scenarios or rare diseases. When analyzed based on the subject matter of questions, the model’s performance was fairly consistent across Medicine, Pediatrics, Obstetrics and Gynecology, and Surgery. This suggests that the model has been successful in acquiring a broad knowledge base in diverse medical fields, supporting its utility as a comprehensive learning tool. The integration of AI into medical education, as noted by Davenport and Kalakota [12], has significant potential to enhance learning experiences by providing personalized and real-time feedback [13]. The present study reaffirms this potential by illustrating ChatGPT’s capability to navigate a variety of medical topics with high accuracy. Furthermore, the utilization of AI in answering SMLE questions opens the door for its application in other standardized medical exams, which would be an exciting avenue for future research. This study sheds light on the effective use of ChatGPT-4 as an AI-assisted educational tool in medical settings. With continuous refinement and training, ChatGPT-4 could revolutionize medical education and exam preparation, providing a valuable resource for students and professionals alike. Based on the findings of our study, we recommend additional research to further refine and evaluate the performance of ChatGPT-4. Training the model with a broader set of questions, including open-ended and scenario-based questions, would potentially enhance its applicability in real-world medical education. Furthermore, expanding the scope of disciplines for training and evaluation would provide a more holistic picture of the model’s capabilities. Lastly, exploring the use of ChatGPT-4 in other standardized medical examinations would validate its generalizability and utility in broader educational contexts.

Our study does have certain limitations. First, our study trained ChatGPT-4 using a limited number of SMLE questions. Increasing the volume of questions for training might improve the model’s performance. Second, while our study included scenario-based MCQs, it did not incorporate other formats like open-ended or short-answer questions. Therefore, our findings may not be fully generalizable to assessments that use diverse question formats. Third, we evaluated the performance of ChatGPT-4 on questions from only four medical disciplines. Including questions from other medical and surgical sub-specialties could provide a more comprehensive understanding of the model’s capability.

## Conclusions

This study evaluated the performance of ChatGPT-4, an AI language model, in answering SMLE questions. The study involved a set of 220 SMLE questions encompassing four medical disciplines, namely, Medicine, Pediatrics, Obstetrics and Gynecology, and Surgery. Statistical analyses using R software revealed that the overall accuracy of ChatGPT-4 in answering the questions was 88.6%. An error analysis identified areas where the model could benefit from further refinement. Here are some potential pathways for refinement: Focused Training on Complex Scenarios: The model’s reduced accuracy with *Hard* questions suggests it may benefit from exposure to more complex and nuanced medical datasets. By training ChatGPT with a higher proportion of intricate clinical scenarios, it may become better attuned to understanding and answering challenging questions. Incorporation of Feedback Loops: Implementing a feedback mechanism where medical professionals can review and correct the model’s outputs, especially on the *Hard* questions it gets wrong, can help it learn from its mistakes. Over time, this iterative feedback can help the model improve its accuracy in identifying the nuances of tougher questions. The model demonstrated high accuracy with *Easy* and *Average* questions but had reduced accuracy with *Hard* questions. Analysis of the model’s performance by subject matter indicated consistent efficacy across all four disciplines. The significance of this study extends beyond merely quantifying the performance of an AI language model in answering medical examination questions. In the field of medical education, there is a growing need for innovative, scalable, and time-efficient assessment tools, particularly for formative assessments that facilitate learning in addition to measuring performance. Our study’s implications are broad and relevant for both medical students and educators. For students, ChatGPT-4 can act as a real-time, supplementary self-assessment tool that helps identify both strengths and areas for improvement. For educators, the model offers a highly accurate and efficient means to design and validate assessments. While promising, we recommend additional studies and refinements before this technology is widely incorporated into medical education and assessments. Its high accuracy in *Easy* and *Average* questions could be leveraged to create baseline assessments, freeing educators to focus on designing more complex and nuanced questions to challenge their students. The study opens a promising avenue for further research into how AI-assisted tools can be effectively integrated into medical education and assessment practices. While our findings indicate strong potential, we recommend additional refinement and validation before the model can be widely adopted in educational settings.
